# Liberation of GPI-Anchored Prion from Phospholipids Accelerates Amyloidogenic Conversion

**DOI:** 10.3390/ijms140917943

**Published:** 2013-09-03

**Authors:** Shen-Jie Lin, Kun-Hua Yu, Jhih-Ru Wu, Chin-Fa Lee, Cheng-Ping Jheng, Hau-Ren Chen, Cheng-I Lee

**Affiliations:** 1 Department of Life Science, National Chung Cheng University, Min-Hsiung, Chia-Yi 621, Taiwan; E-Mails: gonkasim@gmail.com (S.-J.L.); ykhuna@gmail.com (K.-H.Y.); chengpingkevincheng@gmail.com (C.-P.J.); biohrc@ccu.edu.tw (H.-R.C.); 2 Department of Chemistry, National Chung Hsing University, Taichung 402, Taiwan; E-Mails: sana18077@gmail.com (J.-R.W.); cfalee@dragon.nchu.edu.tw (C.-F.L.)

**Keywords:** GPI, cholesterol, phospholipids, misfolding, amyloid fibril, TEM

## Abstract

Prion diseases or transmissible spongiform encephalopathies are a rare group of fatal neurodegenerative illnesses in humans and animals caused by misfolding of prion protein (PrP). Prion protein is a cell-surface glycosylphosphatidylinositol (GPI)-anchored glycoprotein expressed mostly in the central and peripheral nervous system, and this membrane-bound protein can be cleaved from the cell membranes by phosphoinositide phospholipase C. Numerous studies have investigated GPI-free recombinant PrP, but the role of GPI on misfolding of PrP is not well known. In this study, we synthesized a GPI analog that was covalently linking to a PrP S230C mutant, resulting in S230C-GPI. The structural changes in S230C-GPI upon binding to lipid vesicles composed of mixtures of the zwitterionic lipid (POPC) and the anionic lipid (POPG) were analyzed by circular dichroism spectroscopy, and the amyloid aggregation of S230C-GPI in the liberation from phospholipid vesicles was monitored by proteinase K-digestion assay. Our results indicate that S230C-GPI in the liberation of lipid vesicles has high tendency to misfold into amyloid fibrils, while the membrane-bound S230C-GPI proteins are highly stable and rarely convert into amyloid forms. In addition, the role of cholesterol in S230C-GPI was studied. The effect of GPI, cholesterol and phospholipid vesicles on misfolding of PrP is further discussed.

## 1. Introduction

Prion diseases or transmissible spongiform encephalopathies (TSEs) are a rare group of fatal neurodegenerative illnesses in humans and animals. Some TSEs include Creutzfeldt–Jakob disease (CJD), Kuru, Gerstmann–Sträussler–Scheinker (GSS) syndrome, and fatal familial insomnia (FFI). Conversion of the native, predominantly α-helical conformation of prion protein (PrP, PrP^C^) into the β-stranded scrapie isoform of PrP^C^ (PrP^Sc^) is characteristic of TSEs. PrP^Sc^ potentially aggregates into amyloid fibrils having protease resistance.

PrP^C^ is a cell-surface glycosylphosphatidylinositol (GPI)-anchored glycoprotein, expressed mostly in the central and peripheral nervous system [1]. Studies on the localization of PrP in neuroblastoma cells have indicated that both PrP^C^ and PrP^Sc^ exist in lipid rafts [2], especially in caveolae [3]. Caveolae are cavelike invaginations of the cell surface enriched with caveolin, cholesterol, and negatively-charged sphingolipids [4]. Formation of PrP^Sc^ is inhibited when the GPI anchoring is inhibited by deletion or replacement of the C-terminal anchor region of PrP^C^ [5,6]. GPI-anchored prion proteins can be cleaved from cell membranes by phosphoinositide phospholipase C (PI-PLC), but are inaccessible to PI-PLC digestion after structural conversion into PrP^Sc^ [7]. As these results suggest, caveolae provide a place not only for the GPI anchoring of PrP^C^ but also a location for its subsequent conversion to PrP^Sc^.

Caveolae are rich in cholesterol. Thus, the role of cholesterol in PrP^c^ to PrP^Sc^ conversion is also of interest. It has been observed that formation of PrP^Sc^ is inhibited when caveolae are disrupted by cholesterol depletion [5]. The coat proteins of caveolae have a tight association with cholesterol to govern the biochemical behavior of caveolae and signal transduction [8]. Membranes composed of negatively-charged phospholipids are known to induce rapid formation of amyloid fibrils by various proteins [9,10]. However, in one study, the presence of cholesterol actually reduced the stability of the membrane-penetrant form of the amyloid β (Aβ) peptide [11], a peptide whose fibrillation has been implicated in Alzheimer’s disease.

Numerous works have studied GPI-free recombinant PrP. In scrapie-infected transgenic mice expressing anchorless prion protein, abnormal protease-resistant PrP^Sc^ is deposited significantly as amyloid plaque [12]. Anchorless recombinant prion proteins can proceed to PrP^Sc^ in both mouse [13] and cell free systems [14,15]. Recombinant prion protein consists of three α-helices and two antiparallel β-strands between residues 125 and 230 [16]. Partially unfolded recombinant PrP can potentially form β-sheet-rich PrP^Sc^-like fibrils [17,18] that have resistance to proteinase K digestion and that are neurotoxic to transgenic mice [13]. However, the role of GPI on misfolding of PrP is less known. In one study, myristoylation at the *C*-terminus of the prion protein as a GPI anchor mimic indicated that the nonpolar substitution partially impairs amyloid fibril formation, but prion fibrils propagated from myristoylated prion proteins retain similar solvent accessibility [19].

It is important to investigate factors that can potentially affect the structural conversion from PrP^C^ to PrP^Sc^. In the present work, a model was developed based on a single point mutation from serine to cysteine in the *C*-terminal portion of prion protein that allowed for the introduction of a molecule with a long phospholipid chain. Using this model, we were able to obtain new insights on the structural effect of long phospholipid chains in the *C*-terminus of the prion protein.

**Figure 1:**
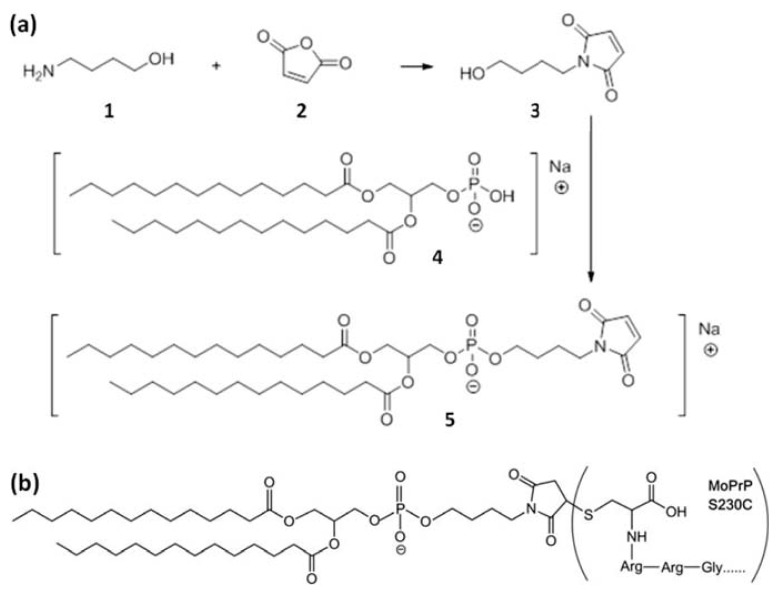
(**a**) Synthesis of GPI-analog molecule; (**b**) The structure of S230C-GPI analog used in this study.

In this study, a molecule containing characteristics of lipids, dubbed GPI analog, was synthesized (Figure 1a). The binding of GPI-analog to mouse PrP (MoPrP) S230C, which resulted in S230C-GPI, was studied with fluorescence and circular dichroism (CD) spectroscopy. The structural change of S230C-GPI upon binding to lipid vesicles composed of the zwitterionic lipid, 1-palmitoyl-2-oleoyl-*sn*-glycero-3-phosphocholine (POPC) and the anionic lipid, 1-palmitoyl-2-oleoyl-*sn*-glycero-3-phospho-1′-rac-1-glycerol (POPG), at a ratio of 3:1 was analyzed. In addition, the effect of cholesterol on MoPrP was investigated. The amyloid forms of S230C-GPI liberated from phospholipid vesicles were imaged by transmission electron microscopy (TEM) and analyzed.

## 2. Results and Discussion

Research on anchorless recombinant prion proteins is abundant, but the effect of long phospholipid chains in GPI-anchored prion proteins is not clear. To study the effect of a GPI anchor on the prion protein, we first synthesized compound **5**, which we dubbed GPI analog, according to the synthetic pathway shown in Figure 1a. GPI analog can covalently bond to cysteine in the *C*-terminus of MoPrP S230C to form a stable carbon-sulfur bond as shown in Figure 1b. Therefore, we have a representative system to study the effect of GPI in folding of prion proteins.

**Figure 2:**
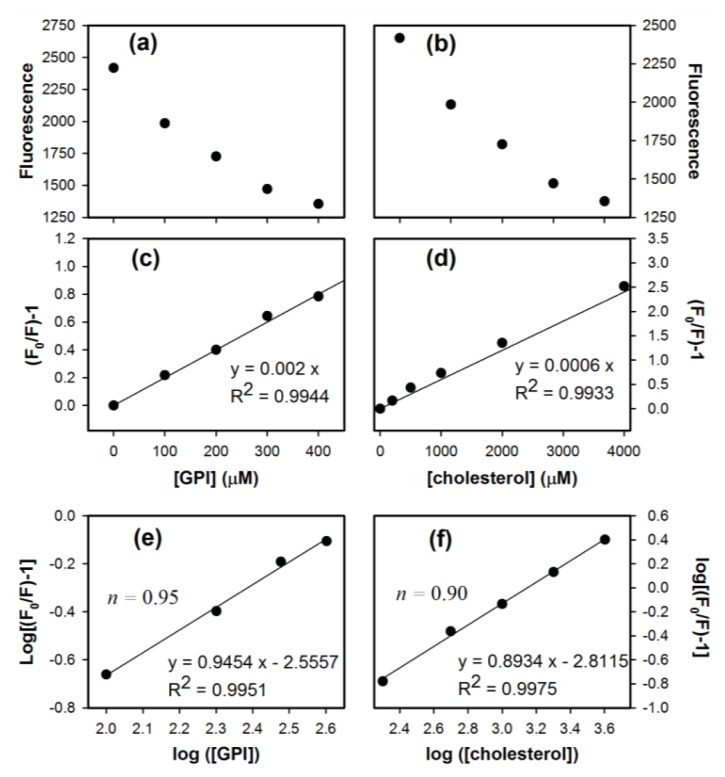
Titration of (**a**) GPI-analog into 10 μM S230C; and (**b**) cholesterol into 10 μM WT monitored by fluorescence spectroscopy; Plots (**c**) and (**d**) are Stern–Volmer analysis based on the data in (**a**) and (**b**), respectively; (**e**) and (**f**) are plots of log [(F_0_/F) − 1] as a function of log ([GPI]) and log ([cholesterol]), respectively, to determine the number of binding site(s).

To investigate the binding of GPI-analog with MoPrP S230C and interaction of cholesterol with MoPrP, we conducted experiments of fluorescence titration as shown in Figure 2a,b. The fluorescence quenching of tryptophan can be monitored by emission intensity at 350 nm upon titration of ligands (GPI-analog or cholesterol) into proteins and described by Stern–Volmer equation as shown in Equation 1 [20].

(1) $$\frac{F_{0}}{F}=1+K_{SV}[Q]$$

where F_0_ and F are the fluorescence intensity without and with the quencher, respectively. *K_SV_* is the Stern-Volmer quenching constant, and [*Q*] is the concentration of the quencher. A purely static quenching process would exhibit a good linear relationship. According the results illustrated in Figure 2c,d, linear relation of (F_0_/F) − 1 *vs*. GPI and (F_0_/F) − 1 *vs*. cholesterol indicates the static quenching process in S230C and MoPrP, respectively. The binding event can be further analyzed. The number of ligand binding sites (*n*) can be determined from Equation 2 for the static quenching.

(2) $$\text{log }\left(\frac{F_{0}-F}{F}\right)=\text{log}K_{a}+n\text{log}[Q]$$

where *K*_a_ is the binding constant for ligand–MoPrP interaction. Linear regression analyzed in Figure 2e revealed that one MoPrP S230C protein binds to about one GPI-analog as expected, since MoPrP S230C has only one covalent linkage to GPI-analog at the *C*-terminus. In the same way, the binding site of cholesterol is calculated to be 0.9, very close to one, as determined in Figure 2f.

**Figure 3:**
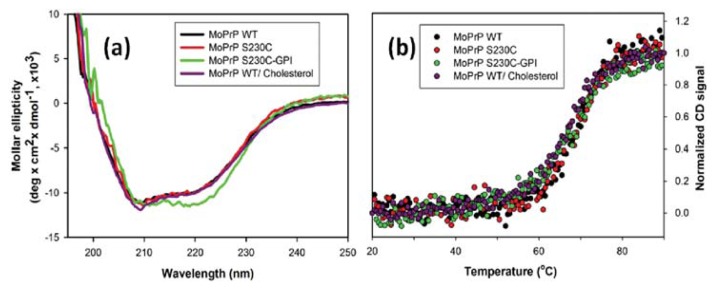
Structure analysis by CD spectroscopy. (**a**) CD spectra and (**b**) thermal-denaturation of 10 μM prion proteins including WT, S230C and S230C-GPI in MES buffer (pH 6), and WT in 50 μM cholesterol containing MES buffer (pH 6).

After confirmation of interaction of S230C with GPI and MoPrP with cholesterol, the protein structure resulting from attachment of the ligands was determined by circular dichroism (CD) spectroscopy. As compared in Figure 3a, the secondary structures of MoPrP WT and MoPrP S230C were identical with significant α-helical conformation represented by the signature at 208 and 222 nm, indicating that this single-point mutation does not result in structural alteration. When MoPrP was exposed to cholesterol, the secondary structure was not modified either. Similarly, linkage of GPI analog to MoPrP S230C results in a structure that was α-helical. This linkage enhanced the α-helical signal at 222 nm slightly. MoPrP contains three α-helices located at the *C*-terminus. Thus, mild modification caused by attachment of GPI analog at the *C*-terminus was expected. Interestingly, this structural modification enhances rather than weakens the α-helical conformation.

A noteworthy structural effect of GPI and cholesterol is the stability of α-helical structure. The structural stability of the S230C mutant, S230C-GPI and MoPrP-cholesterol were determined by heat-induced denaturation monitored at 222 nm as shown in Figure 3b. Consistently, the melting point of MoPrP WT, S230C, S230C-GPI and MoPrP-cholesterol was determined at ~68 °C based on Gibbs–Helmholtz equation. Therefore, none of the single-point mutation, GPI attachment, and cholesterol interaction altered the structural stability.

**Figure 4:**
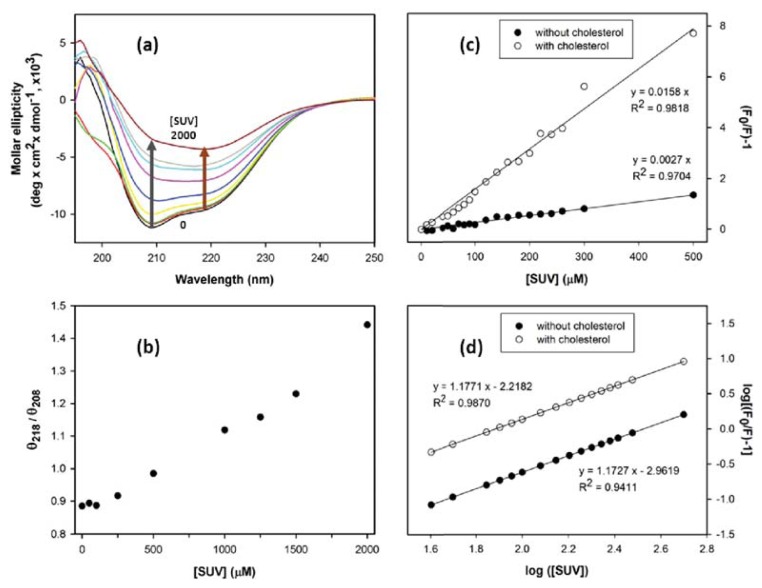
Titration of SUV into S230C-GPI monitored by CD and fluorescence spectroscopy. (**a**) CD spectra of 10 μM S230C-GPI in MES buffer (pH 6) titrated with SUV at concentration of 0, 50, 100, 250, 500, 1000, 1250, 1500, and 2000 μM; and (**b**) determination of β-sheet content represented by the signal ratio of θ_218_/θ_208_; (**c**) Stern-Volmer plot based on the titration of SUV into 10 μM S230C-GPI in the absence and in the presence of 2 mM cholesterol. The signal was monitored by fluorometer at 350 nm; The fluorescence data were further analyzed for binding affinity in (**d**).

Studies using model lipid membranes have revealed that binding of PrP^C^ to lipid membrane via a GPI anchor induces intermolecular β-sheet structure [21,22]. We obtained CD spectra of S230C-GPI upon titration with SUV (Figure 4a). As the α-helical and β-sheet structures can be represented by the ellipticity at 208 and 218 nm, respectively, the extent of β-sheet structure can be estimated by the signal ratio of θ_218_/θ_208_ (Figure 4b). Consistent with previous work using phosphatidylinositol [23] and POPG as lipid membranes [24], our titration experiment indicated that SUV significantly induced formation of β-sheet structure when the concentration was above 500 μM (PrP:SUV > 1:50).

Furthermore, we are interested in the interaction of S230C-GPI and lipid membranes in the presence of cholesterol since cholesterol is rich in lipid membranes. The interaction of S230C-GPI and lipid membranes was determined by fluorescence spectroscopy of S230C-GPI on titration of SUV in the absence and in the presence of cholesterol. The fluorescence intensity of tryptophan at 350 nm was recorded along the SUV titration for Stern–Volmer analysis (Figure 4c). Clearly, the straight lines indicate that interaction of SUV with S230C-GPI goes through a purely static quenching process arising from specific binding between SUV and S230C-GPI. This result is consistent with a previous investigation in which anionic POPG was bound to MoPrP and where subsequent incorporation of low level of zwitterionic POPC slightly weakened the binding [24]. The quenching effect is affected by cholesterol as expected. However, cholesterol does not alter the number of proteins binding to SUV because the number of binding sites was determined to be one, regardless of the amount of cholesterol present (Figure 4d). The SUV-bound MoPrP determined in this study is only one. This low number of bindings could be due to the small membrane surface (radius ~10 nm) in the SUVs prepared in this work. Our CD results revealed significant structural conversion upon SUV binding, suggesting that the binding is close to the native α-helical conformation in the *C*-terminus. The binding region of S230C-GPI to SUV is further discussed in the later section for Figures 6 and 7.

**Figure 5:**
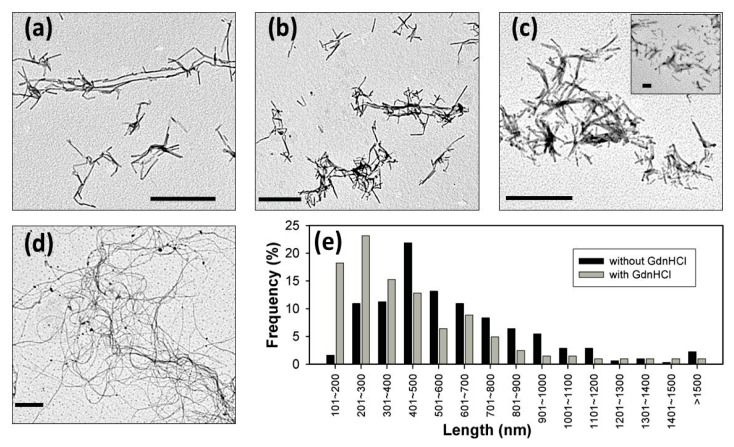
TEM images of prion fibrils converted from (**a**) WT with 2 M GdnHCl; (**b**) S230C-GPI with 2 M GdnHCl; (**c**) S230C-GPI without GdnHCl; (**d**) WT with 2 mM cholesterol; and (**e**) Statistics of the length distribution of S230C-GPI fibrils. The scale bars in (**a**)–(**d**) represents 1 μm.

We are able to consistently observe the fibrils converted from WT PrP, as shown in Figure 5a. Typically, recombinant PrP converts into fibrillar form in the presence of denaturants, such as urea or GdnHCl [17,18]. In the absence of denaturants, recombinant PrP is extremely stable with α-helical structures and does not spontaneously form the PrP^Sc^-like fibrillar form. Like recombinant PrP, the α-helical S230C-GPI converts into amyloid fibrils in the presence of 2 M GdnHCl (Figure 5b). However, unexpectedly, S230C-GPI also converted into the amyloid form in the absence of GdnHCl (Figure 5c) after overnight incubation at room temperature. The fibrils formed in the absence of GdnHCl are long (average 558 nm) and normally distributed, while the fibrils grown with 2 M GdnHCl are relatively short (average 393 nm) and widely-distributed, as compared in Figure 5e.

Under physiological conditions, PrP can be cleaved from the membrane surface by PI-PLC. This cleavage product can be represented by S230C-GPI. The spontaneous conversion of S230C-GPI to amyloid fibrils observed in this work suggests that when the chaperone proteins, or folding assistants, are partially or fully dysfunctional, PrP^C^ might spontaneously convert to PrP^Sc^ in the physiological environment. In addition, overnight incubation of MoPrP in cholesterol containing buffer induced formation of long and curved fibrils as illustrated in Figure 5d. This is a strong indication that high cholesterol plays a significant role in PrP^Sc^ formation.

**Figure 6:**
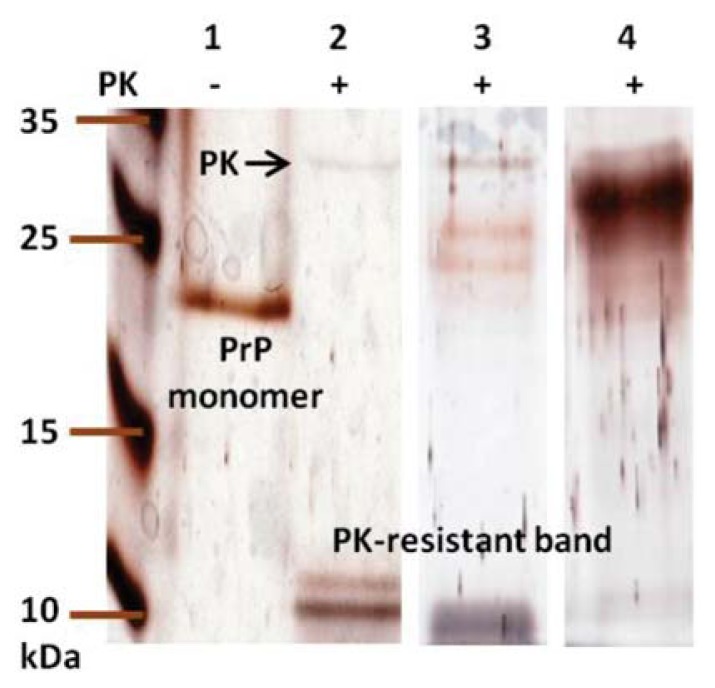
PK-digestion of the amyloid form of MoPrP analyzed by SDS-PAGE followed by silver staining. Lane 1 represents the fibrils converted from WT without PK-treatment. Lanes 2, 3, and 4 are PK-treated fibril samples converted from WT, S230C-GPI and S230C-GPI/SUV, respectively. Lanes 1–2 and lanes 3–4 were run in two independent experiments.

One special feature of PrP^Sc^ is that PrP^Sc^ is resistant to digestion by proteinase K (PK). Similar to PrP^Sc^, prion fibrils are resistant to PK digestion at the *C*-terminal regions. PK-resistant bands with molecular weights 8, 10, and 12 kDa have been clearly identified in amyloid fibrils converted from recombinant MoPrP [15]. In Figure 6, we observed that PK treatment of the amyloid forms of MoPrP WT resulted two small PK-resistant fragments at ~10 kDa (lane 2), whereas only residual MoPrP monomers (23 kDa) appeared in the absence of PK (lane 1). For S230C-GPI fibrils analyzed in another trial, a wide band rising from PK-resistance at ~10 kDa appeared (lane 3). This dark band might be an overlap of two PK fragments. When S230C-GPI was exposed to SUV in a ratio of 1:1 (PrP:SUV), S230C-GPI/SUV monomer distributed broadly, possibly due to the difference in size of the lipid fragments disrupted after SDS-treatment (lane 4).

**Figure 7:**
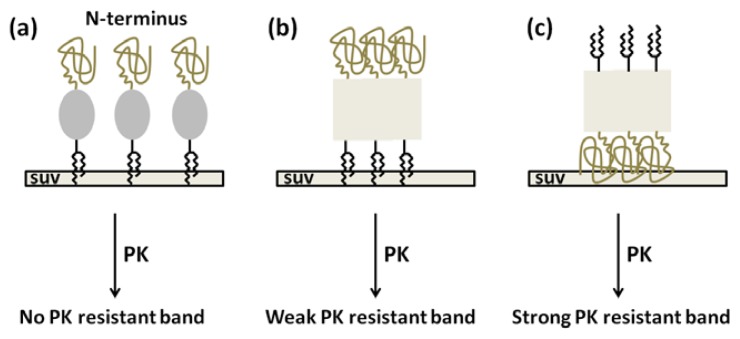
Representative illustration of possible binding of S230C-GPI to SUV. (**a**) The *C*-terminal region of S230C-GPI binds to SUV and S230C-GPI remains natively α-helical folded. PK resistant band will not be observed; (**b**) The *C*-terminal region of S230C-GPI binds to SUV. Prion proteins aggregate on the lipid vesicles. This aggregation weakens the accessibility of PK to the amyloid region located in the *C*-terminus, so the PK resistant band is weak; (**c**) The *N*-terminus of S230C-GPI binds to SUV and the proteins aggregates. Exposed amyloidogenic *C*-terminus can be digested by PK resulting strong PK resistant band.

In contrast to other PK-resistant patterns, abundant S230C-GPI/SUV monomer rather than PK-resistant fragments were observed. This phenomenon indicates that S230C-GPI proteins are highly stable when inserted into lipid vesicles and barely convert to amyloid forms. This result is different from the extensive survey on the lipid-induced amyloid formation of prion in which the PK-resistant fragments in the mixture of POPC and anionic lipid was reported [24]. The inconsistency could be due to the linkage of GPI-analog to S230C and the low dose of lipid vesicle used in this study. In addition, the binding of S230C-GPI to the lipid vesicle should be carried out in the *C*-terminal region rather than the *N*-terminus as illustrated in Figure 7. A study using atomic force microscopy suggested that prion proteins aggregate on the zwitterionic and anionic membranes [25]. The aggregation on POPC, the zwitterionic membranes, does not disrupt the lipid bilayer, while the presence of anionic membranes results in sponge-like aggregates that are disruptive to the membranes. Based on the study with atomic force images, S230C-GPI proteins might aggregate on the SUV as representative by Figure 7b,c. In the case of *N*-terminal binding of PrP to lipid vesicles as illustrated in Figure 7c, the structural unchanged PrP would be digested by PK completely, whereas the amyloid forms in the *C*-terminal region would become highly accessible for PK digestion resulting strong PK resistant band. In the case of *C*-terminal binding of PrP to lipid vesicles, if proteins aggregate as shown in Figure 7b, PK resistant band should be observed. Since the ratio of PrP:SUV used in this experiment has no structural effect as determined in CD spectra shown in Figure 4a, the S230C-GPI should still be α-helically-folded, as represented by Figure 7a. The α-helical PrP is PK-digestible, so the diverse distribution of PrP monomer in lane 4 should represent truncated PrP in addition to the different size of lipid fragments. Eventually, the GPI-anchored PrP on the lipid vesicle is resistant to amyloid formation. Our result is consistent with a previous study indicating the inability of PrP to transform into PrP^Sc^ in a cell-free system, where PrP is associated with raft membranes until the GPI anchor is cleaved [26]. Our result is also consistent with the recent animal study suggesting GPI-knockout lowers the activation energy barrier of pathological propagation [27].

GPI-lipid anchoring is an important post-translational modification in eukaryotes. GPI-anchored proteins are functionally diverse. They are involved in functions including signal transduction, cell–cell interaction, cell adhesion, and host defense [28]. Among the lipid anchors of membrane proteins, GPI anchors have the most unique structures containing oligosaccharides and inositol phospholipids. In this study, our GPI analog contains a long phospholipid chain but lacks oligosaccharides. Our CD results suggest that a long phospholipid chain in the GPI anchor has no structural effect on MoPrP. However, the structural effect of oligosaccharides on MoPrP conformation requires further investigation.

Clustering of GPI-anchored proteins on the membrane microdomain of the cell surface is cholesterol-dependent [29,30]. Cellular cholesterol is a required factor for signal transduction via GPI-anchored proteins beyond the formation of membrane domains [31]. Our CD analysis indicates that a low cholesterol level keeps MoPrP in the natively-folded, α-helical structure. However, when an excess amount of cholesterol is added, a part of the α-helix structure of MoPrP is converted into β-sheet structure. This change promotes the amyloid formation as shown by our TEM images and potentially causes the loss of physiological functions. This result is consistent with the studies related to Alzheimer’s disease indicating that high cholesterol levels are significantly tied to brain plaques associated with Alzheimer’s disease [32,33], and studies related to diabetes [33]. Partial unfolding is a critical step in amyloid formation, especially at the middle point of unfolding [34]. Our previous protein simulation suggested that the structure of PrP is partially α-helical in the *C*-terminus in the native condition [35] and at the melting temperature [36]. It is known that amyloid fibrils contain a rigid hydrophobic core axially [37], and a high cholesterol level might stabilized the hydrophobic interaction between partially folded PrP monomers resulting in cross β-sheet structures in the amyloid form.

## 3. Experimental Section

### 3.1. Synthesis of GPI-Analog

Briefly, compounds **1** and **2** were mixed in acetic acid and heated to 170 °C for 6 h. Compound **3** was formed after returning the solution to the room temperature [38,39]. Subsequently, 1, 2-dimyristoyl-*sn*-glycero-3-phosphate (compound **4**) was dissolved in pyridine and warmed to 35 °C and added with 2,4,6-triisopropylbenzenesulfonyl chloride. Reaction of compounds **3** and **4** at 35 °C for 4.5 h yielded compound **5** [40]. At the end of synthesis, the solvent was removed under reduced pressure and the crude product was purified by chromatography and identified by GC–MS.

### 3.2. Covalent Linkage of MoPrP S230C and GPI Analog

Experimentally, the mixture of MoPrP and GPI analog at ratio of 1:3 was incubated in a cold room overnight. The S230C-bound, and free GPI analog, were separated by HPLC equipped with a reverse-phase C4 column (Waters) and eluted with gradient of water and acetonitrile. The formation of a covalent linkage between MoPrP S230C and GPI analog was confirmed by 16% Tricine SDS-PAGE [41].

### 3.3. Plasmid Construction

S230C single point mutation was introduced into MoPrP 23-230 gene using QuickChange^®^ XL site-directed mutagenesis kit (Agilent Technologies, Santa Clara, CA, USA) with primers:

Forward: 5′-GAAGA**TGT**TAAAAGGGCGAGCTCAAGGC-3′

Reverse: 3′-GGATAATGCTGCCCTCTTCT**ACA**TTATTC-5′

S230C plasmid obtained from PCR was confirmed by DNA agarose gel and the sequence of S230C plasmid was further confirmed by sequence analysis.

### 3.4. Protein Expression and Purification

The plasmids pET101 encoding MoPrP 23-230 (WT) and S230C were transformed into competent *Escherichia coli* BL21 star™ (DE3, Life Technologies, Grand Island, NY, USA) cells respectively, in 50 mL LB medium overnight. In large-scale culture, cells were transferred to TB medium and grew until optical density at 600 nm reached 0.6. Subsequent addition of 1 mM isopropyl β-d-1-thiogalactopyranoside induced expression of proteins in inclusion body. The proteins were purified by immobilized metal affinity chromatography and reversed-phase C4-HPLC as previously described procedure [17,42]. The purified MoPrP was confirmed by SDS-PAGE to be a single species with correct molecular weight.

### 3.5. Preparation of Small Unilamellar Lipid Vesicles (SUV)

In the preparation of SUV, 1-palmitoyl-2-oleoyl-*sn*-glycero-3-phosphocholine (POPC) and 1-palmitoyl-2-oleoyl-*sn*-glycero-3-phospho-1′-rac-1-glycerol (POPG) both purchased from Avanti Polar Lipids were preliminarily dissolved in chloroform. Mixture of POPC and POPG at the molar ratio of 3:1 was dried in a test tube. Subsequently, the lipids were dissolved with distilled water and sonicated at 40 °C for 40 min. The sonicated solution was quickly frozen with liquid nitrogen for 20 min, and then heated to 40 °C for 20 min. This freezing and heating cycle was repeated 5 times. Finally, the lipids were sonicated for 20 min followed by centrifugation at 12,000 rpm in eppendorf tubes. The desired SUV was populated in the supernatant. The radius of SUV was about 10 nm as determined by dynamic light scattering (Nano Zeta, Malvern, UK).

### 3.6. Fluorescence Spectroscopy

GPI and cholesterol solutions were titrated into 10 μM MoPrP S230C and WT, respectively, in 50 mM MES buffer (pH 6.0). The spectra of tryptophan residues in the proteins upon each titration were collected by Hitachi F-4500 fluorometer with excitation wavelength at 280 nm. The fluorescence intensity at 350 nm was recorded for further analysis.

### 3.7. Circular Dichroism Spectroscopy

CD spectra of MoPrP samples were recorded with a Jasco J-815 spectrometer. For measurements in the far-UV region, a quartz cell with a path length of 0.1 cm was used in nitrogen atmosphere. For protein samples, the concentration was kept constant at 10 μM in 20 mM MES (pH 6.0). An accumulation of five scans, with a scan speed of 50 nm per minute, was performed at 20 °C. The heat-induced denaturation of proteins was conducted with heating protein solutions at the rate of 1 °C/min, and the ellipticity at 222 nm was collected every 0.5 °C.

### 3.8. Fibril Conversion

For fibril conversion, proteins were added into 50 mM MES (pH 6) in the absence or in the presence of 2 M GdnHCl. The samples were incubated at 37 °C, as described in previous studies on protein fibrillation [17,18]. When the ThT fluorescence reading reached the steady state, the fibril samples were dialyzed with H_2_O for further experiments.

### 3.9. Transmission Electron Microscopy

The fibril samples were stained with 2% tungsten phosphoric acid onto carbon-coated 200-mesh copper grids. The samples were adsorbed onto the copper grids for 30 s and subsequently washed with PBS and H_2_O twice. The samples were air-dried overnight before imaging. The TEM images were collected by a Joel JEM-2100 TEM. The analysis of fibril length was carried out on WICF ImageJ software (National Institutes of Health, Bethesda, MA, USA).

### 3.10. Proteinase K Digestion

The amyloid fibrils (10 μM) were dialyzed and treated with PK (0.2 μM) at 37 °C for 1 h in 100 mM Tris–HCl buffer (pH 7.2). This PK-digestion was stopped by addition of urea to a final concentration of 2.25 M. Samples were heated at 95 °C for 10 min and analyzed by 12.5% SDS-PAGE followed by analysis with silver staining. In addition to the silver staining, aliquots of PK-treated samples were analyzed with mass spectroscopy for precise molecular weight.

## 4. Conclusions

GPI-anchoring on lipid vesicles keeps PrP in native structure and with normal function. Liberation of PrP from membrane surface potentially converts normal PrP^C^ into disease-related amyloid form, PrP^Sc^. Enhancement of linking PrP on the membrane domain can be a direction to develop approaches to prevent and to alleviate TSE.
